# What Is the Survival Rate of Implants Loaded Immediately with a Branemark Protocol Prosthesis? A Review

**DOI:** 10.1055/s-0044-1787818

**Published:** 2024-07-16

**Authors:** Josuel Siqueira Azarias, Victor Augusto Alves Bento, Clóvis Lamartine de Moraes Melo Neto, Manuel Martin Adriazola Ique, Daniela Micheline do Santos, Marcelo Coelho Goiato

**Affiliations:** 1 Department of Dental Materials and Prosthodontics, School of Dentistry, São Paulo State University (UNESP), Araçatuba, São Paulo, Brazil

**Keywords:** dental, prosthesis, implant-supported prostheses, temporary, restoration, immediate implant loading

## Abstract

The aim of this review was to determine the survival rate of implants loaded immediately with a Branemark protocol prosthesis. An electronic search was performed in the PubMed/MEDLINE database from 2006 to February 2024, using a combination of Medical Subject Headings descriptors: “completely edentulous” and “immediate loading.” Human clinical articles in English that evaluated the survival rate of implants loaded immediately with a Branemark protocol prosthesis after placement in the bone were included. A total of 546 articles were identified, of which 15 were included in this study. The 15 articles included in the literature showed a high implant survival rate (more than 93%) after immediate loading.

## Introduction

The loss of all permanent teeth (edentulism) 1 impairs the individual's chewing function, nutrition, speech, esthetics, and quality of life. 2 The types of treatment that can rehabilitate individuals with this oral condition can be treated with conventional full dentures and implant-supported prostheses (overdentures [removable] and Branemark protocol prostheses [screw-retained]). 3 4 5 6 7 8 9 10 11 12 13 14 15 16 17 18

Treatment with implants and implant-supported prostheses is considered the gold standard in dentistry. 19 After implants are placed in edentulous patients, a period of several months is required for osseointegration (4 months for the mandible and 6 months for the maxilla), which is defined as the neoformation of bone around the implant. 16 19 20 As a result, the individual's oral function temporarily worsens, causing dissatisfaction. 16 In this context, in order to achieve “immediate” function, it is possible to consider the immediate loading of implants with a Branemark protocol prosthesis. Currently, the term “immediate implant loading” refers to a procedure that requires the implant to be loaded immediately or within 48 hours of its placement. 12 21 This type of treatment allows for immediate prosthesis usability, improved appearance, and high patient satisfaction. 15 21 However, the immediate loading of implants with a Branemark protocol prosthesis depends on their number and the level of primary stability achieved.

Normally, for the maxilla and mandible, a minimum of six and five implants, respectively, are needed to install a Branemark protocol prosthesis. However, with the “All-on-four” concept, only four implants can be used to support this type of prosthesis. 13 The placement of four implants, two posterior implants tilted distally (which allows longer implants to be placed) and two vertical implants in the anterior region (“All-on-four” concept), allows bone grafting procedures to be avoided and reduces the number of implants, which leads to lower treatment costs. 13 22

Achieving adequate primary stability is a prerequisite for osseointegration. 19 Primary stability is represented by intimate initial contact between the bone and the implant immediately after its placement in the bone. 19 23 The level of initial contact between bone and implant can determine the possibilities for loading the implant (immediate, early, or late). 24 Also, this level of initial contact must stop the implant from moving very slightly above 150 μm, whether it is loaded or not right away, so that the implant does not get wrapped up in fibrous tissue and fail to fuse with the bone. 23 24 For immediate loading of a single implant, the recommended insertion torque is 45 N·cm (high primary stability), 24 but for Branemark protocol prostheses, the implant insertion torque can be moderate (30–44 N·cm). 24 25

The aim of this literature review was to determine the survival rate of implants loaded immediately with a Branemark protocol prosthesis.

## Methods

An electronic search was carried out in the PubMed/MEDLINE database from 2006 to February 2024, using a combination of descriptors from the Medical Subject Headings: “completely edentulous” and “immediate loading.” Human clinical articles were included, in English, that evaluated the survival rate of implants loaded immediately with a Branemark protocol prosthesis after their placement in the bone. When information on marginal bone loss around the implants was available in the article, it was collected.

## Results

The search identified a total of 546 articles in PubMed/MEDLINE. This study included 15 articles that met the inclusion criteria out of the total. 5 7 8 9 10 11 12 13 14 15 16 17 18 26 27 The implant survival rate was high in all the included articles (above 93%). Table 1 describes the characteristics of the included studies.

**Table 1:** Studies included in the literature review

Author (year)	Type of study	Number of patients	Number of implants	Implant bed	Number of implants per arch	Waiting period for the prosthesis to be installed after surgery	Follow-up	Implant survival rate (%)	Was immediate loading viable?
Maló et al (2003) 5	Retrospective	44	176	Edentulous mandibles	All-on-four	On the same day of surgery	3 y	>96	Yes
Testori et al (2008) 7	Prospective	41	164	Edentulous maxillae	Six implants	48 h	5 y	>97	Yes
Bergkvist et al (2009) 8	Prospective	28	168	Edentulous maxillae	Six implants	24 h	3 y	98.2	Yes
Li et al (2009) 9	Retrospective	111	690	Edentulous maxillae and mandibles	Average of 6.65 implants in the maxilla and 4.36 implants in the mandible	On the same day of surgery	5.9 y	98.7	Yes
Covani et al (2012) 10	Prospective	19	164	Edentulous maxillae and mandibles	Six implants in the mandible and/or eight implants in the maxilla	48 h	4 y	95.1	Yes
Maló et al (2015) 11	Retrospective	43	172	Edentulous maxillae	All-on-four	On the same day of surgery	3 y	>95	Yes
Shigehara et al (2015) 12	Prospective	27	189	Edentulous maxillae and mandibles	Average of 7 implants in the maxilla and 5.6 implants in the mandible	On the same day of surgery	5 y	100	Yes
Gherlone et al (2018) 13	Prospective	29	128	Edentulous maxillae and mandibles	All-on-four	On the same day of surgery	5 y	>98	Yes
Kim et al (2018) 14	Retrospective	26	370	Edentulous maxillae and mandibles	Average of 8.11 implants in the maxilla and 6.12 implants in the mandible	On the same day of surgery	7 y	96	Yes
Windael et al (2018) 15	Prospective	25	125	Edentulous mandibles	Five implants	24 h	10 y	100	Yes
Kaneda et al (2019) 16	Retrospective	52	220	Edentulous mandibles	Four–six implants based on the All-on-four concept	On the same day of surgery	10 y	93.9	Yes
Eskan et al (2020) 17	Retrospective	42	171	Edentulous maxillae and mandibles	Four implants per arch	On the same day of surgery	4.5 y	97.7	Yes
Werbelow et al (2020) 18	Retrospective	23	170	Edentulous maxillae and mandibles	Four–six implants per arch	On the same day of surgery	9 y	100	Yes
Carosi et al (2021) 26	Retrospective	37	160	Edentulous maxillae and/or mandibles	Four implants per arch	On the same day of surgery	4 y	96.9	Yes
Mohamed et al (2022) 27	Prospective	20	80	Edentulous mandibles	Four implants	On the same day of surgery	1 y	100	Yes

## Review

### Maló et al (2003)

In the study by Maló et al (2003), 5 the “All-on-four” technique was used with Brånemark System implants (Nobel Biocare AB) with a length of 10 to 18 mm and a diameter of 3.75 to 4.0 mm. All implants were installed with a torque of more than 40 N·cm in the anterior region of the edentulous mandible. In this study, there were cases in which implants were installed in the socket immediately after tooth extraction (12 patients) and cases in which implants were installed in healed bone (32 patients). The lower corner of the implant neck was aimed at bone level, and whenever possible, bicortical anchorage was established.

The screw-retained acrylic provisional prostheses were reinforced with a metal strip and delivered within 2 hours of implant placement surgery. Subsequently, the definitive prostheses were installed 4 to 6 months after surgery. The provisional prostheses were made without a cantilever, whereas the definitive prostheses were made with a small cantilever.

The implant survival rate after 6 months was 96.7%. Subsequently, from 6 months to 1 year, from 1 to 2 years, and from 2 to 3 years, implant survival rates were 100%. The survival rates for provisional and definitive prostheses were 100%. Marginal bone levels, assessed on periapical or panoramic radiographs, were recorded at the last follow-up appointment within the study period. The bone level was, on average, 1.2 mm below the abutment–implant interface.

### Testori et al (2008)

In the study by Testori et al (2008), 7 each patient received four axial implants and two distal inclined implants in the edentulous maxilla (Osseotite NT Implant, 3i Implant Innovations). The insertion torque of the implants was 30 N·cm or more. In most of the cases, the implant shoulder was placed at the crest. All of the posteriorly tilted implants required bone contouring on the distal aspect, allowing for proper seating of the prosthesis.

For each patient, a provisional acrylic prosthesis screwed on with provisional metal cylinders was delivered within 48 hours of the implant placement surgery. The final prosthesis was delivered 3 months later. The presence or absence of a cantilever was not reported in this study.

The overall cumulative implant survival rate was 97.9 and 97.1% for axially positioned and tilted implants, respectively, up to 3 years of observation. These same percentages were maintained after 5 years of follow-up. No prosthetic failures occurred, resulting in an overall prosthetic success rate of 100%. Radiographic assessment of the change in marginal bone level was carried out after 1 year. Crestal bone loss averaged 0.9 ± 0.4 and 0.8 ± 0.5 mm for axial and inclined implants, respectively, at the 12-month assessment. No significant difference was recorded in the change in bone level between the two implant groups.

### Bergkvist et al (2009)

In the study by Bergkvist et al (2009), 8 the technique of six implants was used in the edentulous maxilla with lengths ranging from 10 to 12 mm and diameters of 4.8, 4.1, and 3.3 mm (regular neck, Straumann AG). The average implant stability quotient (ISQ) immediately after implant placement was 50.6. No cases of immediate tooth extraction with immediate implant installation in the socket were reported. The implant platform was positioned 1 to 2 mm below the bone crest.

Screw-retained acrylic provisional prostheses without cantilevers were made from self-curing acrylic resin and delivered 24 hours after implant placement. The provisional fixed prostheses were in use for an average of 15 weeks, after which the definitive prostheses were installed. The definitive prostheses had cantilevers.

Mean marginal bone loss from baseline to 8 months after loading was 1.6 mm ( *p* = 0.094), from 8 to 20 months 0.41 mm ( *p* = 0.094), and from 20 to 32 months 0.08 mm ( *p* = 0.039). The 32-month cumulative survival rate was 98.2%. There were no losses of provisional or definitive prostheses, as all prosthetic complications were resolved.

### Li et al (2009)

In the study by Li et al (2009), 9 an average of 6.65 implants were installed in the maxilla and 4.36 implants in the mandible. The types of implants varied: Brånemark System MK III, which were externally connected parallel implants; Brånemark System MK IV/NobelSpeedy, which were externally connected parallel implants with an enlarged cone, specially designed for soft bone; Replace Select Tapered/NobelReplace Tapered Groovy, which were internally connected tapered implants; Replace Select Straight/NobelReplace Straight Groovy, which were internally connected parallel implants. The length of the implants varied from 7 to 18 mm, and the platforms used also varied (narrow, regular, and wide). Maximum insertion torque values were recorded for each implant during surgery, ranging from 20 to 50 N·cm. Extractions and immediate implantations were sometimes carried out. Implants and grafts were often placed simultaneously. After installing the implants, their platform remained at the level of the bone crest.

Screw-retained acrylic provisional prostheses were attached to titanium cylinders connected to the implants immediately after surgery. A definitive implant impression at the abutment level was taken after 6 weeks of soft tissue healing, and the definitive prostheses were finished within 3 months. The presence or absence of cantilevers was not reported in this study.

The immediate loading protocol constituted cumulative survival rates of 98.7% for the maxilla and 98.7% for the mandible, with an overall cumulative survival rate of 98.7%. The mean marginal bone loss was found to be 0.07 mm after 1 year.

### Covani et al (2012)

In the study by Covani et al (2012), 10 patients received six implants each in the mandible and/or eight maxillary implants (implants with a bioceramic, Ossean, Intra-Lock International) with an insertion torque of 45 N·cm or more. The study presented situations in which the implants were installed in the healed bone and situations in which the implants were installed in the alveoli after tooth extractions.

For each patient, a screw-retained acrylic prosthesis was installed 48 hours after surgery. The presence or absence of cantilevers was not reported in this study.

The 4-year cumulative survival rate was 95.1%. All the failed implants were placed in mature bone. The authors did not report results on prosthesis failure rates and marginal bone loss.

### Maló et al (2015)

In the study by Maló et al (2015), 11 the “All-on-four” technique was used with NobelSpeedy conical implants (external hexagon) with lengths ranging from 7 to 18 mm and a diameter of 4.0 mm, installed with a torque of between 35 and 50 N·cm in the maxilla. The implant neck was aimed at being positioned at bone level, and bicortical anchorage was established whenever possible, using the maxillary crest and the nasal corticals in the anterior region for the short-length implants. Implants were installed in the healed bone.

For each patient, a temporary acrylic prosthesis screwed on with titanium cylinders was fabricated in the dental laboratory and installed on the same day as surgery. Anterior occlusal contacts and the orientation of the canines during lateral movements were preferred in the provisional prosthesis. The definitive prostheses were installed after 6 months. The presence or absence of cantilevers was not reported in this study.

Three short and three long implants failed in four patients, resulting in a cumulative overall implant and patient survival rate, respectively, of 95.7 and 95.1% for short implants, 100% for regular implants, and 96.6 and 95.2% for long implants. Mechanical complications were recorded in 13 patients (30%) between 2 and 36 months of follow-up: 7 provisional prostheses fractures and 6 abutment screw loosening. The fractures were mended, and the abutments were retightened, followed by the readjustment of the occlusion. All situations remained stable throughout the follow-up of the study. The average marginal bone remodeling at 1 and 3 years was 0.97 and 1.25 mm for short implants, 0.82 and 0.87 mm for regular implants, and 0.87 and 0.98 mm for long implants.

### Shigehara et al (2015)

In the study by Shigehara et al (2015), 12 an average of 7 implants were installed in the maxilla and 5.6 implants in the mandible (Straumann implants, SLA) with an insertion torque of at least 35 N·cm. Straumann Standard/Plus or Tapered Effect implants were chosen, depending on bone quality. The diameter (3.3, 4.1, and 4.8) and length of the implants installed varied (6, 8, 10, and 12 mm).

For each patient, a screw-retained acrylic provisional prosthesis was made from acrylic resin without additional reinforcement materials, such as metal frames. The provisional prostheses were designed without distal cantilevers. The definitive restoration was performed more than 2 months after surgery. Mandibular definitive prostheses had a small cantilever, and maxillary ones did not. For the final restorations, a distal cantilever was avoided in all maxillary cases, and a distal cantilever of one tooth was made when the implants were inserted between the mental foramen in the mandible.

The cumulative survival rate of the implants was 100%, and the success rate of the prostheses was also 100% during the observation time. Although there were mechanical complications with the provisional prostheses (four prostheses fractured), no loss of this type of prosthesis was reported. The success rate of the definitive prostheses was 100%. The authors did not assess marginal bone loss.

### Gherlone et al (2018)

The study by Gherlone et al (2018) 13 used the “All-on-four” technique, and the implants (Winsix implants, Biosafin) were placed in one or both arches. The posterior implants were 4.5 mm in diameter and 15 or 13 mm long, while the anterior implants were 4.5 or 3.8 mm in diameter and 13 mm in length. The insertion torque ranged from 30 to 40 N·cm. The study did not report any cases in which an implant was placed in the socket immediately after tooth extraction.

For each patient, a screw-retained acrylic resin provisional prosthesis reinforced with a metal bar was fabricated. The cantilevers were extended to the first molar region and, in three cases, only to the second premolar. The final prostheses were placed at 4 months, but the presence or absence of cantilevers in them was not reported.

The overall 5-year survival rate of the implants was 100% for the axially placed implants and 98.44% for the obliquely placed implants. Implant survival rates were 100% in the maxilla and 98.75% in the mandible. None of the 32 fixed prostheses were lost during the observation period, resulting in a prosthesis survival rate of 100%. At the 60-month evaluation, mean peri-implant crestal bone loss was 1.08 mm for upright maxillary implants ( *n* = 24 implants) and 1.02 ± 0.67 mm for tilted maxillary implants ( *n* = 24 implants). In the mandible, the mean peri-implant crestal bone loss was 1.04 mm for upright implants ( *n* = 40) and 1.09 mm for angled implants ( *n* = 40). There were no statistically significant differences in crestal bone loss between tilted and upright implants at the 6-, 12-, and 60-month follow-up evaluations in either jaw.

### Kim et al (2018)

At the time of implant placement, approximately 12.7 teeth were extracted per patient, and more than 52% of all 370 (194 implants) were immediately placed. 14 An average of 8.11 implants were installed in the maxilla and 6.12 in the mandible (Osstem Implant Co., Ltd., Busan, Korea, and Dentium Co.). The implants installed were of the internal connection type, with a diameter of approximately 3.4 to 7.0 mm for the maxilla and 3.0 to 7.0 mm for the mandible. For both the maxilla and mandible, the implants were between 8.0 and 14.0 mm long. The insertion torque was 50 N·cm.

For each patient, a provisional removable complete denture was placed in the maxilla, and a screw-retained acrylic provisional denture was installed in the mandible immediately after the implant placement surgery. Provisional fixed prostheses were made using cylindrical titanium abutments and self-curing acrylic resins. The presence or absence of cantilevers was not reported in this study. The 7-year cumulative survival rate of immediate loading was 96%. The authors did not evaluate marginal bone loss.

### Windael et al (2018)

In the study by Windael et al (2018), 15 the technique of installing five implants in the mandible in the region between the foramina was used. The implant width (3.5–5 mm) and length (8–15 mm) were chosen by the surgeon based on bone quantity and quality. The shoulder of each implant was completely surrounded by bone. There were no reports of tooth extractions followed by implant installation.

For each patient, the implants were loaded with an acrylic screw-reinforced provisional prosthesis 1 day after surgery. After 3 months, the provisional bridge was removed, and osseointegration was assessed by tightening the abutments with 20 N·cm. The presence or absence of cantilevers was not reported in this study.

None of the implants nor the connected prosthesis was lost during the 10-year follow-up, resulting in a 100% survival rate. Bone loss was calculated by comparing periapical radiographs taken during recall appointments after 3, 1, 2, and 10 months. The average bone loss around the implants after 3, 12, 24, and 120 months was 0.16, 0.14, 0.17, and 0.49 mm, respectively.

### Kaneda et al (2019)

In the study by Kaneda et al (2019), 16 four to six implants were installed in fully edentulous mandibles (NobelSpeedy Groovy, Brånemark System Mark III or Mark IV, NobelReplace tapered; diameter, 3.3–4.3 mm; length, 7–15 mm; Nobel Biocare, Japan) with at least 30N of primary stability. The implants were placed following the All-on-four concept. It is not clear from the article whether or not implants were installed in tooth sockets left after tooth extractions.

For each patient, a screw-retained acrylic provisional prosthesis was installed on the day of surgery, and no cantilever was present. Only anterior occlusal contacts were used in the provisional prosthesis, and there was no posterior occlusal contact for 2 months. After 2 months, acrylic resin was added to the posterior region of the provisional prosthesis to obtain occlusal contacts. A secondary acrylic provisional prosthesis was then fitted at least 3 months after surgery. Subsequently, the definitive prostheses were made. It was not reported whether the definitive prostheses had cantilevers.

Thirteen implants in seven patients failed, and the 10-year cumulative implant survival rate was 93.9%. The prosthesis survival rate was 100%. This article did not assess bone loss around the implants.

### Eskan et al (2020)

In the study by Eskan et al (2020), 17 the BLT implant system (Straumann BLT SLA Roxolid Basel), which has regular and narrow-diameter implants, was used. The length (8, 10, 12, 14, and 16 mm) and diameter (3.3 and 4.1 mm) of the implants varied. Four implants were installed in the edentulous maxilla or mandible, with the exception of two cases, in one of which five implants were installed in the mandible and in the other, six implants were installed in the maxilla. The insertion torque of the implants was at least 30 N·cm. There were cases in which the implants were installed in healed bone and cases in which the implants were installed in fresh alveoli.

Screw-retained acrylic provisional prostheses attached to temporary titanium copings were delivered on the day of surgery and had no cantilevers. The maximum cantilever on each side of the definitive prostheses was no more than 10 to 12 mm.

Four implants (three implants in the maxilla and one implant in the mandible) were lost, resulting in an overall cumulative implant survival rate of 97.7%. Around 15% of the provisional acrylic prostheses were broken during the healing period in the first 4 months, but they were repaired. The final prosthesis survival rate was 100%. The mean interproximal marginal bone loss was 0.15 mm after 24 months. The mean of the bone loss from 0 to 24 months was 0.15 mm, and the mean of the bone loss between 12 and 24 months was 0.09 mm.

### Werbelow et al (2020)

In the study by Werbelow et al (2020), 18 four to six implants were installed in the maxilla and/or mandible (blueSKY implants, Bredent GmbH & Co. KG, Senden, Germany), with an insertion torque of at least 30 N·cm. Tooth extraction and smoothing of the bone surfaces were carried out, if necessary, prior to the placement of four to six dental implants.

Screw-retained acrylic provisional prostheses were installed 2 to 2.5 hours after implant placement surgery. The definitive prostheses were installed 3 to 6 months after surgery. The presence or absence of cantilevers was not reported in this study.

There were no implant failures or losses during the 6- to 9-year follow-up period (100% success rate). During the follow-up period, 3 of the 23 patients had a resin tooth fracture. The fractured teeth of the three patients were repaired within 1 to 2 hours by the dental technician. No significant changes in marginal bone levels were recorded after the 6- to 9-year follow-up period in any patient. No bone crest loss was detected at any of the implant sites.

### Carosi et al (2021)

In the study by Carosi et al (2021), 26 four implants (Ø ≥4.1 mm or Ø 3.3 mm and lengths of 8, 10, 12, and 14 mm) were installed in the maxilla and/or mandible. The types of dental implants were: bone-level tapered regular crossfit, bone-level tapered narrow crossfit, and bone-level regular crossfit. Insertion torque was at least 35 N·cm. Carosi et al (2021) did not report whether implants were installed in sockets after tooth extraction.

Screw-retained acrylic temporary prostheses with titanium copings were installed on the day of the implant placement surgery. The definitive prostheses were installed at least 3 months after surgery. The provisional prostheses did not have cantilevers. For definitive prostheses, the length of the cantilevers in the mandible was limited to 15 mm, and in the maxilla, it was limited to 12 mm.

The overall implant survival rate is 96.9%. No definitive prostheses failed, resulting in a 100% prosthetic success rate. The failed implants were successfully replaced before definitive prostheses were made, resulting in an overall treatment success of 100%. This study did not evaluate marginal bone loss around the implants.

### Mohamed et al (2022)

In the study by Mohamed et al (2022), 27 two groups were created: axial group: received four implants (Dentium superline; Dentium), two placed in the lateral incisor region and the other two in the first molar region (12 mm in length and 3.6 mm in diameter) and tilted group: received four interforaminal implants, two anterior vertically aligned (12 mm in length and 3.6 mm in diameter) and two posterior distally inclined implants (14 mm in length and 3.6 mm in diameter). The mean ISQ values for all implants were above 65. There were no cases of implant placement in the socket after tooth extraction.

Screw-retained acrylic temporary prostheses with metal cylinders were installed on the day of the implant placement surgery. The provisional prostheses did not have cantilevers. The definitive prostheses were installed 3 months after the surgery. Based on definitive prostheses, for the tilted group, a short cantilever was extended posteriorly in the form of one tooth (first molar) with no more than 1.5 times the anteroposterior distance between the anterior and posterior implants, and no cantilever was made for the axial group.

No implant failures were detected, with a success rate of 100%. None of the definitive prostheses fractured throughout the follow-up period (1 year). The highest value for crestal bone loss was 0.89 mm after the first year.

## Discussion

All the articles included in this review showed that immediate loading of implants (up to 48 hours) with a Branemark protocol prosthesis (screw-retained) is a viable treatment due to the high implant survival rate (more than 93%). 5 7 8 9 10 11 12 13 14 15 16 17 18 26 27 This high success rate was observed for both mandibular and maxillary rehabilitations in follow-ups ranging from 1 to 10 years ( Table 1 ). 5 7 8 9 10 11 12 13 14 15 16 17 18 26 27

In all the articles included, all screw-retained provisional prostheses were made of acrylic resin with or without reinforcement. 5 7 8 9 10 11 12 13 14 15 16 17 18 26 27 Therefore, even when fractures in these prostheses were reported, it was possible to repair them. 8 11 12 17 18 It is worth mentioning that, in general, the survival rate of provisional and definitive prostheses was 100%. 5 7 8 11 12 13 15 16 17 18 26 27

The literature reports that the posterior cantilever should have a maximum length of 1.5 times the distance between the most posterior point of the most posterior implant and the center of the most anterior implant in the arch (clinically acceptable). 22 28 Eight articles reported that provisional prostheses had small or absent cantilevers. 5 8 12 13 16 17 26 27 When the provisional prostheses had cantilevers, the calculation to define what the length of the posterior cantilevers should be was not informed. Regarding definitive prostheses, six articles clearly reported that they had cantilevers. 5 8 12 17 26 27 Among these six studies, only the study by Mohamed et al (2022) reported that when cantilevers were present, they had clinically acceptable lengths 27 according to the previously reported rule. 22 28 Therefore, it is recommended that future studies of this nature also report such a calculation so that the reader can know whether the length of the cantilevers was within the clinically acceptable range.

Marginal bone loss in height of up to 2 mm around the implant after the first year of its placement (osseointegration and loading) is considered clinically normal. 29 30 31 Based on this information, all the articles that evaluated marginal bone loss around the implant showed clinically acceptable values (< 2 mm) after 1 year of follow-up. 5 7 8 9 11 13 15 17 18 27

It is possible to interpret the insertion torque value obtained after placing an implant in the bone as follows: (1) insertion torque values < 30 N·cm represent low implant stability; (2) insertion torque values between 30 and 44 N·cm represent moderate implant stability; and (3) insertion torque values ≥ 45 N·cm represent high implant stability. 24 It is reported that moderate primary stability values are sufficient for the immediate loading of implants with a Branemark protocol prosthesis. 25 Eleven of the articles included in this review presented an insertion torque equal to or greater than 30 N·cm. 5 7 10 11 12 13 14 16 17 18 26 Windael et al (2018) did not report insertion torque values 15 ; and Li et al (2009) installed implants with a torque ranging from 20 to 50 N·cm, that is, implants with low and high primary stability were loaded. 9

The primary stability level of an implant, based on ISQ values, can be interpreted as follows: (1) ISQ values < 60 represent low primary stability; (2) ISQ values from 60 to 64 represent medium–low primary stability; (3) ISQ values from 65 to 69 represent medium–high primary stability; and (4) ISQ values ≥ 70 represent high primary stability. 32 Immediate loading of implants with a Branemark protocol prosthesis can be carried out when the ISQ value of the primary stability of the implants is 60 or higher. 32 Based on this information, Bergkvist et al (2009) loaded the implants tested with an insufficient ISQ value (mean value of 50.6); however, the survival rate of the implants 3 years after immediate loading was high (98.2%). 8 32 Mohamed et al (2022) loaded the implants tested with an adequate ISQ value (mean value of 65), and the survival rate of the implants after 1 year of follow-up was also high (100%). 27 32

Based on the articles included, most of the time, the primary stability values (torque and ISQ) obtained ranged from moderate to high, which is clinically safer for long-term implant survival.

## Conclusion

The 15 articles in the literature included in this study showed a high implant survival rate (more than 93%) after their immediate loading with Branemark protocol prostheses.
